# Short-term exposure to ambient air pollutants increases the mortality risk of chronic kidney disease: a time-stratified case-crossover study in Zhejiang Province

**DOI:** 10.1186/s12882-026-05103-9

**Published:** 2026-07-21

**Authors:** Pinyuan Dai, Xiaoyan Zhou, Weiwei Gong, Jin Pan, Yunqi Guan, Hao Wang, Na Li, Jieming Zhong

**Affiliations:** Zhejiang Provincial Center for Diseases Control and Prevention, Hangzhou, Zhejiang 310051 China

**Keywords:** Chronic kidney disease, Air pollutants, Mortality, Time-stratified case-crossover design

## Abstract

**Background:**

The association between air pollutants and mortality from chronic kidney disease (CKD) remains to be further investigated. This study aims to examine the impact of air pollutant exposure on CKD mortality in individuals.

**Methods:**

This study utilized an individual-level, time-stratified case-crossover design to evaluate the association between short-term exposure to air pollutants (PM_2.5_, SO_2_, O_3_, and NO_2_) and mortality from CKD. The deceased population was identified from the mortality surveillance data of the Zhejiang Provincial Center for Disease Control and Prevention. For each decedent, we collected air pollutant concentrations on the day of death (case day) and on matched control days within the same month. This design inherently controls confounding factors such as age, place of residence, and seasonality.

**Results:**

The study found that short-term exposure to all four air pollutants was associated with an increased risk of CKD mortality. The excess risk (ER) per interquartile range (IQR) increase in pollutant concentrations was evaluated across different lag periods. Using multi-day moving average lags (calculated as the mean exposure from the current day up to the specified preceding day), the most prominent ERs were observed for PM2.5 at lag02 (ER: 15.16%, 95% CI: 12.69–17.68), SO_2_ at lag03 (ER: 8.80%, 95% CI: 5.95–11.72), O_3_ at lag01 (ER: 5.67%, 95% CI: 2.07–9.39), and NO_2_ at lag03 (ER: 13.66%, 95% CI: 9.12–18.39). After adjusting for interactions with other pollutants, the associations for SO_2_ and PM_2.5_ remained significant. Additionally, the study found that this association was influenced by factors such as marriage, and season (cold vs. warm).

**Conclusions:**

This study revealed a significant association between short-term exposure to air pollutants and CKD mortality in the population of Zhejiang Province. These findings provide novel evidence for understanding the impact of environmental factors on kidney disease and suggest a need for governmental authorities to enhance the monitoring and control of air pollution to mitigate the health burden on the elderly.

**Supplementary Information:**

The online version contains supplementary material available at https://doi.org/10.1186/s12882-026-05103-9.

## Introduction

Chronic kidney disease (CKD) is a chronic disease characterized by progressive structural damage and functional decline of the kidneys. According to data from the Global Burden of Disease (GBD) study, the global prevalence of CKD has increased by 29.3% and the mortality rate has risen by 41.5% since 1990 [1]. This has resulted in a heavy disease and economic burden on individuals, families, and society. Research suggests that without effective intervention, the number of global deaths from CKD could reach 30 million by 2040 [2]. The growing disease burden is not only reflected in direct medical costs but also leads to a decline in patients’ quality of life and a loss of social productivity, posing a significant challenge to the global health, economic, and social landscape. This rapidly escalating trend and its profound impact on global public health highlight the urgent need to identify modifiable risk factors for CKD and design effective, targeted preventive intervention strategies.

Environmental factors play a crucial role in the onset and progression of CKD, with the hazards of air pollution being one of the primary concerns for researchers today. Common air pollutants can enter the human circulatory system through various routes. As the body’s main excretory organ, the kidney is characterized by high blood flow, a large endothelial surface area, and high metabolic activity. Consequently, during the filtration and concentration process, these pollutants and their metabolites can inevitably cause direct or indirect damage to renal tissue. The potential mechanisms by which air pollutants lead to CKD involve multiple aspects, including oxidative stress, mitochondrial damage, and renal fibrosis. Ambient pollutants can elicit systemic inflammation and oxidative stress, thereby inducing endothelial dysfunction and altering renal hemodynamics. These pathophysiological shifts ultimately contribute to an elevated risk of CKD, the accelerated decline of renal function, and an increased incidence of end-stage renal disease (ESRD) [3]. Therefore, an in-depth investigation into the impact of air pollution on mortality in CKD patients is of great significance for developing effective public health intervention strategies.

However, the existing literature on air pollution and CKD mortality presents critical knowledge gaps. First, while several large-scale cohort studies have established the adverse effects of long-term exposure to fine particulate matter on CKD progression and survival, evidence regarding the short-term, acute triggering effects of daily air pollution fluctuations on CKD mortality remains scarce [4, 5]. Second, current short-term epidemiological studies are predominantly localized in high-income regions and often restricted to small cohorts or specific patient subgroups (e.g., those undergoing dialysis) [6, 7]. This highlights the necessity of conducting a large-scale study on CKD mortality. By exploring the association between different types of air pollutants and CKD mortality, this study seeks to provide new evidence to inform disease prevention and guide the development of public health policies.

## Materials and methods

### Study population

The mortality data used in this study were obtained from the mortality surveillance system established by the Zhejiang Provincial Center for Disease Control and Prevention (Zhejiang CDC). It has been widely used to assess the mortality level in the province and thus holds a certain degree of representativeness [8, 9]. This system employs rigorous quality control procedures, with death certificates reported by professionally trained hospital physicians and subjected to multi-layered review by well-trained coding personnel at local CDCs to ensure coding accuracy. Each death survey record contains all death-related information, including the causal chain of death (encompassing both direct and indirect causes of death) as well as the inferred underlying cause of death. Since 2000, codes for causes of death have been categorized in accordance with the International Classification of Diseases, 10th Revision (ICD-10), based on physicians’ diagnoses. In line with the ICD code range for diseases defined by the Global Burden of Disease (GBD) study, the ICD codes of patients with chronic kidney disease included in this study are D63.1, E10.2, E11.2, I12-I13.9, N02-N08.8, N15.0, N18-N18.9, and Q61-Q62.8 [10]. This study collected residential addresses and demographic information of patients whose underlying cause of death was chronic kidney disease, covering the period from January 1, 2019, to December 31, 2022. The demographic information included age, gender, marital status, and date of death. Additionally, we geocoded the address information into latitude and longitude coordinates using the Baidu Maps API (http://lbsyun.baidu.com/) [11].

### Exposure and other covariates

Pollutant concentration variables in this study were obtained from the China High Air Pollutants (CHAP) dataset (https://weijing-rs.github.io/product.html), a publicly available resource. This dataset provides daily grid average concentrations of NO2 [12], PM_2.5_ [13], SO_2_ [14], and O_3_ [15] at a 1 km × 1 km resolution for Zhejiang Province, covering the period from 2019 to 2022. The ground-level air pollutant data provided by the CHAP dataset boasts advantages of full coverage, long-term availability, seamless, high quality, and high resolution, thus being widely cited in relevant studies. This dataset is generated based on diverse big data sources, including ground-based measurements, remote sensing products, atmospheric reanalysis, and model simulations, using advanced artificial intelligence methods. Meanwhile, the spatiotemporal heterogeneity of air pollution is also taken into account. In addition, the daily average temperature and average humidity data for the 1 km×1 km grid in Zhejiang Province are derived from the fifth-generation European Reanalysis (ERA5)-Land reanalysis dataset [16]. Owing to its comprehensive and consistent atmospheric data, this reanalysis dataset has been widely applied and highly valued in global climate-related studies.

### Study design

For each mortality case due to chronic kidney disease, we employed bilinear interpolation to estimate the concentrations of air pollutants as well as temperature and humidity values at the residential address of the case. This method calculates the weighted average of the four nearest grid points surrounding a location with specific longitude and latitude [17]. Based on these data, a time-stratified case-crossover design was applied at the individual level to analyze the association between CKD mortality and short-term exposure to various pollutants [18]. The time-stratified case-crossover study explores the relationship between exposure and an event by comparing an individual’s exposure levels across different time periods, including the period when the acute event (i.e., death in this study) occurs. It is a widely used epidemiological design, primarily employed to investigate the health impacts of factors such as air pollutants and meteorological conditions. This method controls the case and control periods within the same year, month, and day of week. Therefore, this research design can simultaneously control for confounders, including seasonality, time trends, and day of the week, yielding unbiased parameter estimates [19].

### Statistical analysis

Conditional logistic regression was employed to investigate the short-term effects of environmental air pollutant exposure on chronic kidney disease mortality across different lag periods. The excess risk (ER) and its corresponding 95% confidence interval (CI) were used to quantify the risk level for each pollutant. In this model, the regression coefficient (β) represents the estimated log-odds ratio of mortality associated with a one-unit increase in the specific air pollutant. Specifically, ER represents the percentage increase in chronic kidney disease mortality risk for every interquartile range (IQR) increase in pollutant concentration, calculated as ER = [exp(β × IQR) − 1] × 100%. In practical terms, an ER of 15% indicates a 15% increase in the odds of death for each IQR increment in the respective pollutant. Although our model estimates odds ratios (ORs), the extremely low daily CKD mortality rate satisfies the rare disease assumption. Under this assumption, the OR provides a highly accurate approximation of the relative risk, making our derived ER a reliable measure of true excess risk. In the model, natural cubic spline functions with 5 degrees of freedom were applied to daily mean temperature and relative humidity to adjust for potential meteorological confounding factors. Lag effects were addressed using both single-day lags (representing pollutant concentrations on a specific preceding day) and moving average of multi-day lags (representing the average pollutant concentration over the current day and several preceding days). We evaluated the effects from the current day (lag0) up to seven lag days (lag7), and for moving averages from lag01 to lag07(Supplementary Fig. 1). For example, lag03 represents the mean exposure across the current day and the preceding three days. The lag day with the highest observed effect was selected for baseline comparison and subsequent analyses for each pollutant.

Descriptive statistics for continuous variables were presented as median (interquartile range, IQR), while categorical variables were expressed as counts (percentages, %). Prior to fitting the multi-pollutant models, we assessed potential multicollinearity among the air pollutants by calculating the Variance Inflation Factor (VIF) and conducting correlation analysis. The Mann-Whitney U test or t-test was used to compare differences in pollutant concentrations between different subgroups. Subgroup analyses were conducted based on the following factors: sex, age group (< 75 years and ≥ 75 years), marital status (married vs. others), occupation (farmers vs. others), warm season (May to October) vs. cold season (November to April), and urbanization (urban vs. rural), interaction tests were performed by calculating the Z-score. In addition, three sensitivity analyses were conducted in this study. First, we calculated the corresponding ER by adjusting the degrees of freedom of the daily average temperature and relative humidity parameters in the model, so as to evaluate the stability of the results. Second, air pollutants were divided into two groups according to their median concentrations, and conditional logistic regression was used to calculate the interaction effects of pollutants. The odds ratio (OR) was used to assess the risk level of pollutants after adjusting for the effects of other air pollutants, and the excess risk attributed to the interaction was also quantified. Third, given that our study period (2019–2022) overlapped with the COVID-19 pandemic, we conducted an additional sensitivity analysis to evaluate the potential influence of pandemic-related disruptions. Specifically, we excluded all mortality cases from the year 2022, a period characterized by the relaxation of pandemic control measures and subsequent infections in China, to assess the robustness of our findings against this major time-varying confounder.

All statistical analyses were performed in R version 4.4.0. Two-sided P-values less than 0.05 were statistically significant.

## Result

### Baseline

From 2019 to 2022, a total of 29,340 individuals died from chronic kidney disease in Zhejiang Province, the flowchart is shown in Supplementary Fig. 2. The majority of these deaths occurred in males (53.50%), individuals aged 75 and above (63.13%), those residing in rural areas (60.76%), and during the cold season (55.85%), as shown in Table 1. The median concentrations of air pollutants on case days and control days were 23.25 µg/m³ and 23.71 µg/m³ for PM_2.5_, 7.77 µg/m³ and 7.79 µg/m³ for SO_2_, 25.23 µg/m³ and 25.35 µg/m³ for NO_2_, and 93.00 µg/m³ and 93.15 µg/m³ for O_3_, respectively.

**Table 1:** Characteristics of study population and distribution of four air pollutants

Baseline Characteristic	No. (%)	PM_2.5_ (µg/m^3^)		SO_2_ (µg/m^3^)		NO_2_ (µg/m^3^)		O_3_ (µg/m^3^)	
		Median (IQR)	*P*-value	Median (IQR)	*P*-value	Median (IQR)	*P*-value	Median (IQR)	*P*-value
**Overall**	129,013 (100)	23.35 (16.70-33.05)	< 0.001	7.77 (6.77–9.14)	0.064	25.26 (17.57–36.74)	0.766	93.02 (68.45-122.92)	0.111
Case days	99,673 (77.26)	23.25 (16.65–32.89)		7.77 (6.77–9.13)		25.23 (17.59–36.65)		93.00 (68.32-122.89)	
Control days	29,340 (22.74)	23.71 (16.84–33.68)		7.79 (6.78–9.19)		25.35 (17.50-36.98)		93.15 (69.00-123.04)	
**Sex**			0.262		0.136		0.599		0.123
Male	15,696 (53.50)	23.82 (16.87–33.84)		7.80 (6.79–9.22)		25.38 (17.46–36.90)		92.91 (68.63-122.23)	
Female	13,644 (46.50)	23.51 (16.81–33.49)		7.78 (6.77–9.15)		25.31 (17.54–37.10)		93.36 (69.40-123.96)	
**Age**			0.004		0.057		< 0.001		0.582
<75	10,818 (36.87)	23.38 (16.63–33.09)		7.81 (6.83–9.18)		24.35 (16.98–35.30)		93.57 (69.33-123.13)	
≥75	18,522 (63.13)	23.87 (16.98–33.95)		7.78 (6.74–9.20)		25.89 (17.80-37.91)		92.90 (68.80-122.98)	
**District**			< 0.001		< 0.001		< 0.001		0.534
Urban	11,385 (39.24)	25.02 (17.57–36.25)		7.70 (6.59–9.12)		30.18 (20.76–42.69)		92.89 (66.78–126.90)	
Rural	17,632 (60.76)	22.85 (16.43–31.91)		7.84 (6.89–9.23)		22.51 (15.94–32.48)		93.24 (70.29–120.70)	
**Season**			< 0.001		< 0.001		< 0.001		< 0.001
Cold	16,386 (55.85)	29.89 (22.09–40.96)		8.23 (6.97–9.88)		32.26 (22.64–43.44)		78.01 (58.89-101.26)	
Warm	12,954 (44.15)	18.12 (13.71–23.64)		7.42 (6.63–8.38)		19.35 (14.54–26.11)		114.48 (90.60-141.74)	
**Occupation**			< 0.001		< 0.001		< 0.001		0.280
Farmer	9442 (32.18)	24.35 (16.98–35.26)		7.75 (6.63–9.19)		29.40 (20.08–42.28)		92.62 (67.31-124.45)	
Other	19,898 (67.82)	23.38 (16.78–32.88)		7.81 (6.84–9.19)		23.64 (16.60–34.40)		93.43 (69.65-122.47)	
**Marital status**			0.528		0.019		0.945		0.376
Married	10,208 (34.79)	23.80 (16.99–33.72)		7.75 (6.74–9.14)		25.40 (17.51–37.01)		92.67 (68.32-123.35)	
Unmarried	19,132 (65.21)	23.64 (16.78–33.67)		7.81 (6.80–9.22)		25.32 (17.49–36.96)		93.38 (69.37-122.97)	

Abbreviations: IQR = interquartile range

### Regression results

The VIF values for all pollutants in the multi-pollutant models were less than 3, indicating that multicollinearity was not a significant concern (Supplementary Table 1). Figure 1 illustrates that exposure to PM_2.5_, SO_2_, O_3_, and NO_2_ significantly increased chronic kidney disease mortality in both single-day and moving-average multi-day lag models. The associations between a IQR µg/m³ increase in pollutant concentration and increased chronic kidney disease mortality were observed for PM_2.5_ at lag02 (ER: 15.16%, 95% CI: 12.69–17.68), SO_2_ at lag03 (ER: 8.80%, 95% CI: 5.95–11.72), O_3_ at lag01 (ER: 5.67%, 95% CI: 2.07–9.39), and NO_2_ at lag03 (ER: 13.66%, 95% CI: 9.12–18.39).

**Fig. 1:**
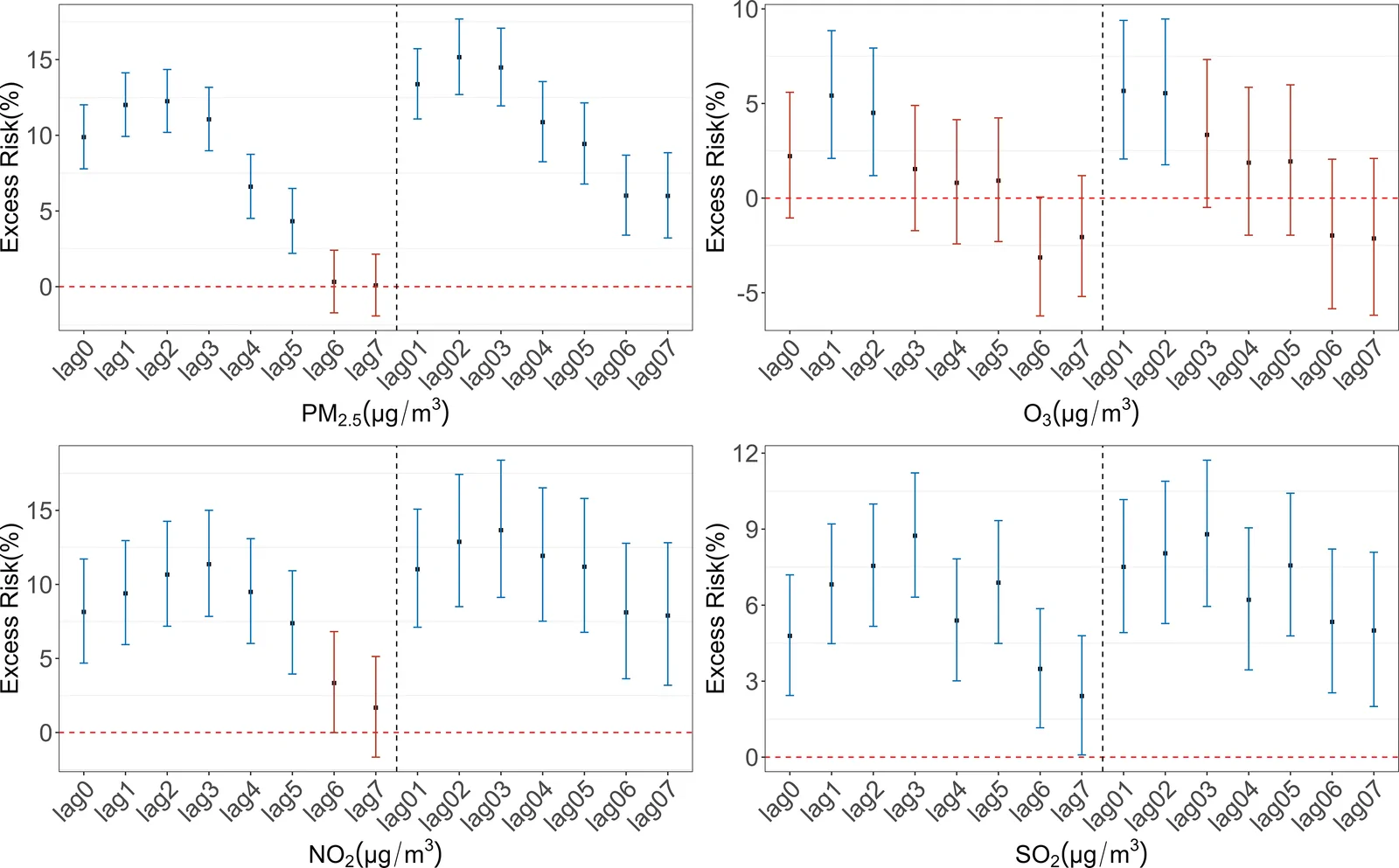
ERs (95% CIs) for CKD mortality associated with each IQR µg/m^3^ increase of exposures to four air pollutants. * The X-axis is the single lag days from lag0 to lag7, and the moving average lag days from lag 01 to lag 07

### Stratified analysis

Figure 2 shows that none of the air pollutants were significantly associated with the mortality rate of chronic kidney disease in the warm season. However, significant associations were observed in the cold season, with the excess risks (ERs) being 16.49%, 15.81%, 15.09%, and 10.04% (*P* < 0.05), respectively. O₃ also exhibited varying results across other subgroups. In contrast, NO₂ and SO₂ did not show significant differences in their associations across subgroups stratified by gender, age, occupation, or residential area (urban vs. rural). Nevertheless, significant correlations were observed in the unmarried population (with ER of 3.38%, *P* < 0.05), while no such associations were found in the married population.

**Fig. 2:**
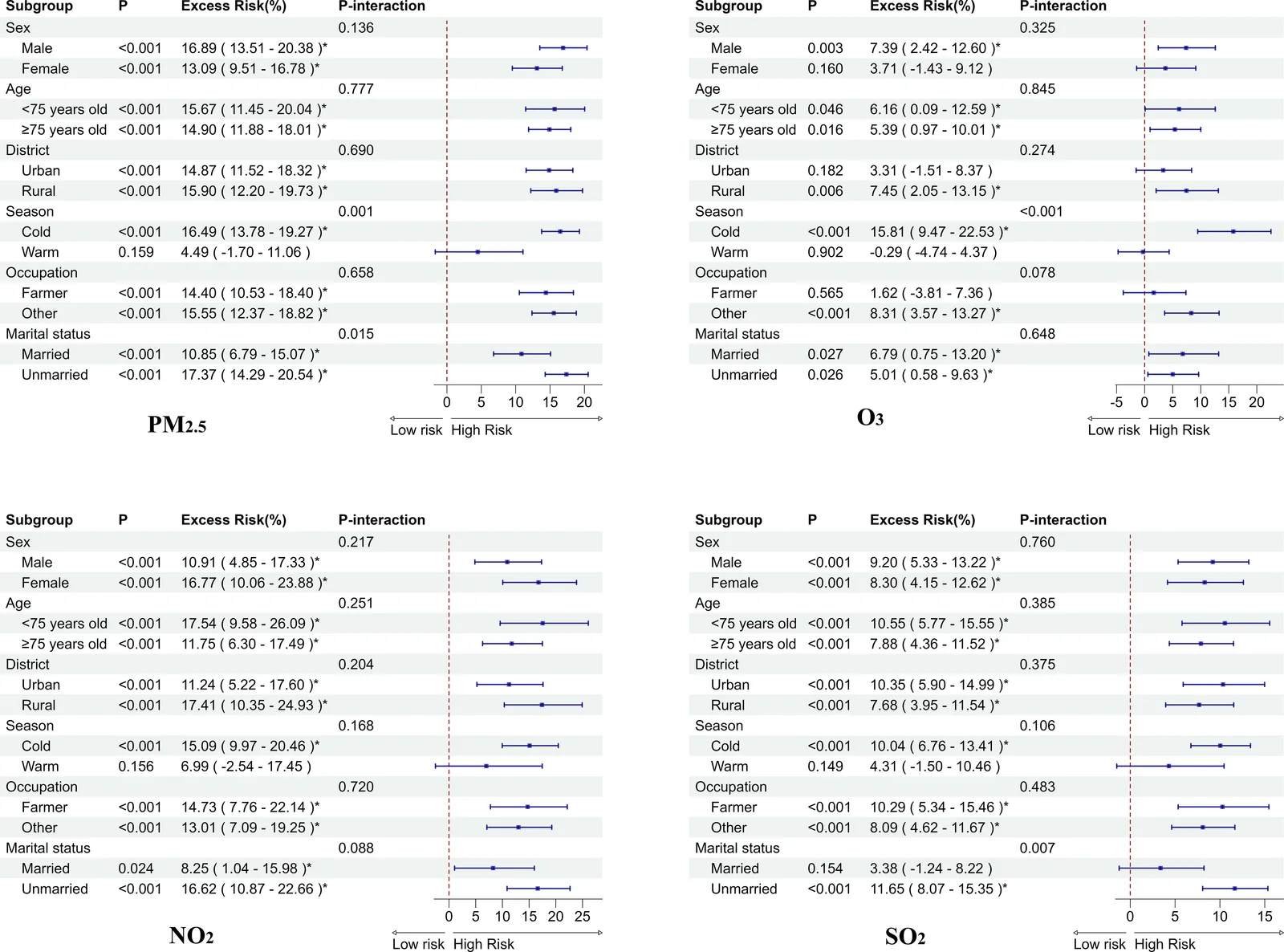
ERs (95% CIs) for CKD mortality associated with each IQR µg/m^3^ increase in exposure to four air pollutants stratified by subgroup

### Sensitivity analysis

As shown in Fig. 3, the association between air pollutant exposure and CKD mortality remained stable after adjusting for the degrees of freedom of temperature and humidity in the model. Supplementary Table 2 summarizes the results of the interaction tests. Significant interaction effects were identified between several pollutant pairs, specifically NO_2_ and SO_2_, NO_2_ and PM_2.5_, with a relative excess risk due to interaction (RERI) of -0.74 (95% CI: -0.84, -0.65) and − 0.80 (95% CI:-0.90, -0.70). Furthermore, as shown in Fig. 4, PM_2.5_ and SO_2_ exposure remained associated with CKD mortality after adjusting for the effects of other pollutants. The sensitivity analysis excluding data from the year 2022 is presented in Supplementary Fig. 3. Although the ER for CKD mortality associated with short-term air pollutant exposure were slightly attenuated compared to the primary analysis, the overall positive associations remained consistent.

**Fig. 3:**
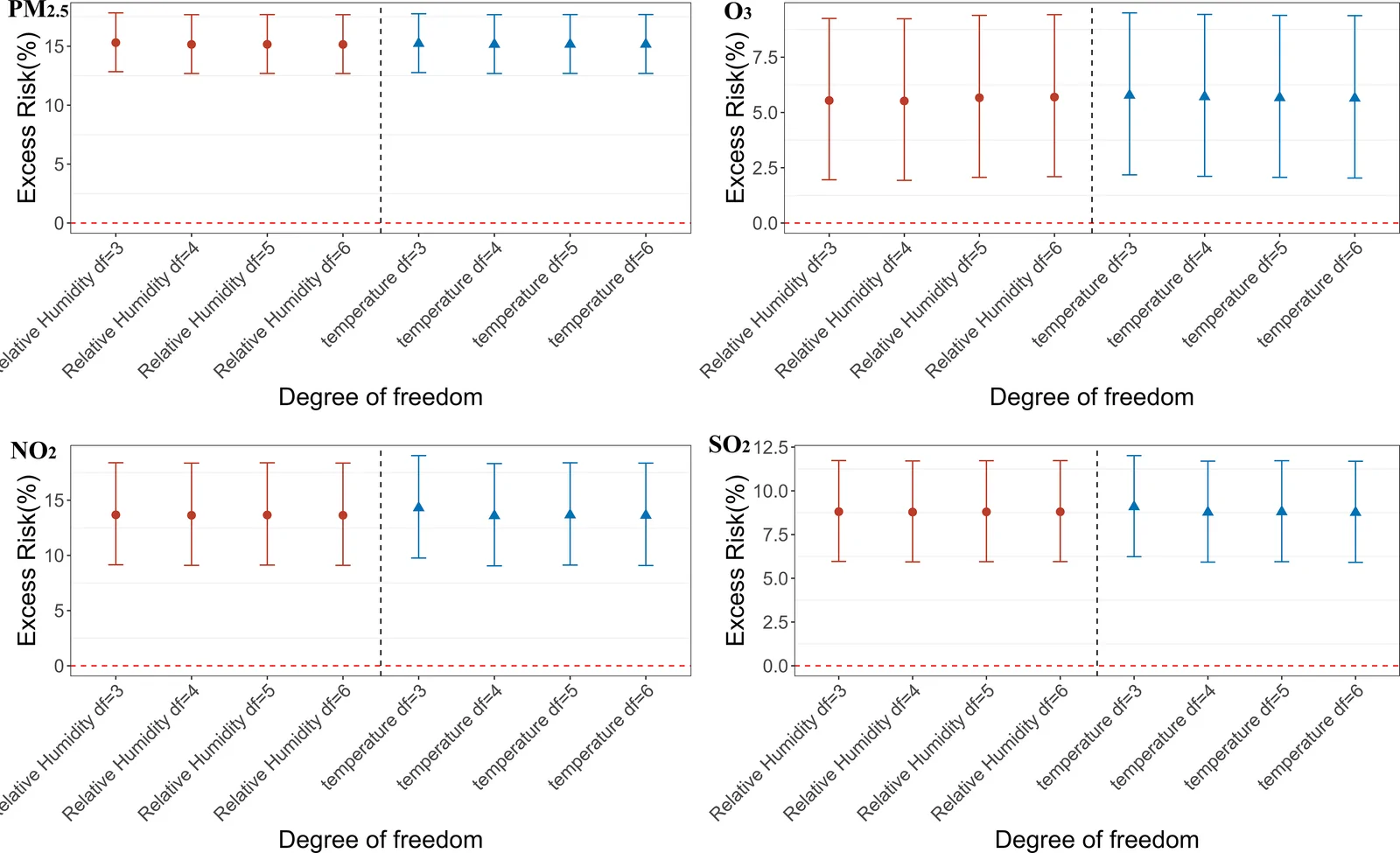
ERs (95% CIs) for CKD mortality associated with each IQR µg/m^3^ increase in exposure to four air pollutants, adjusted for different degrees of freedom for temperature and relative humidity

**Fig. 4:**
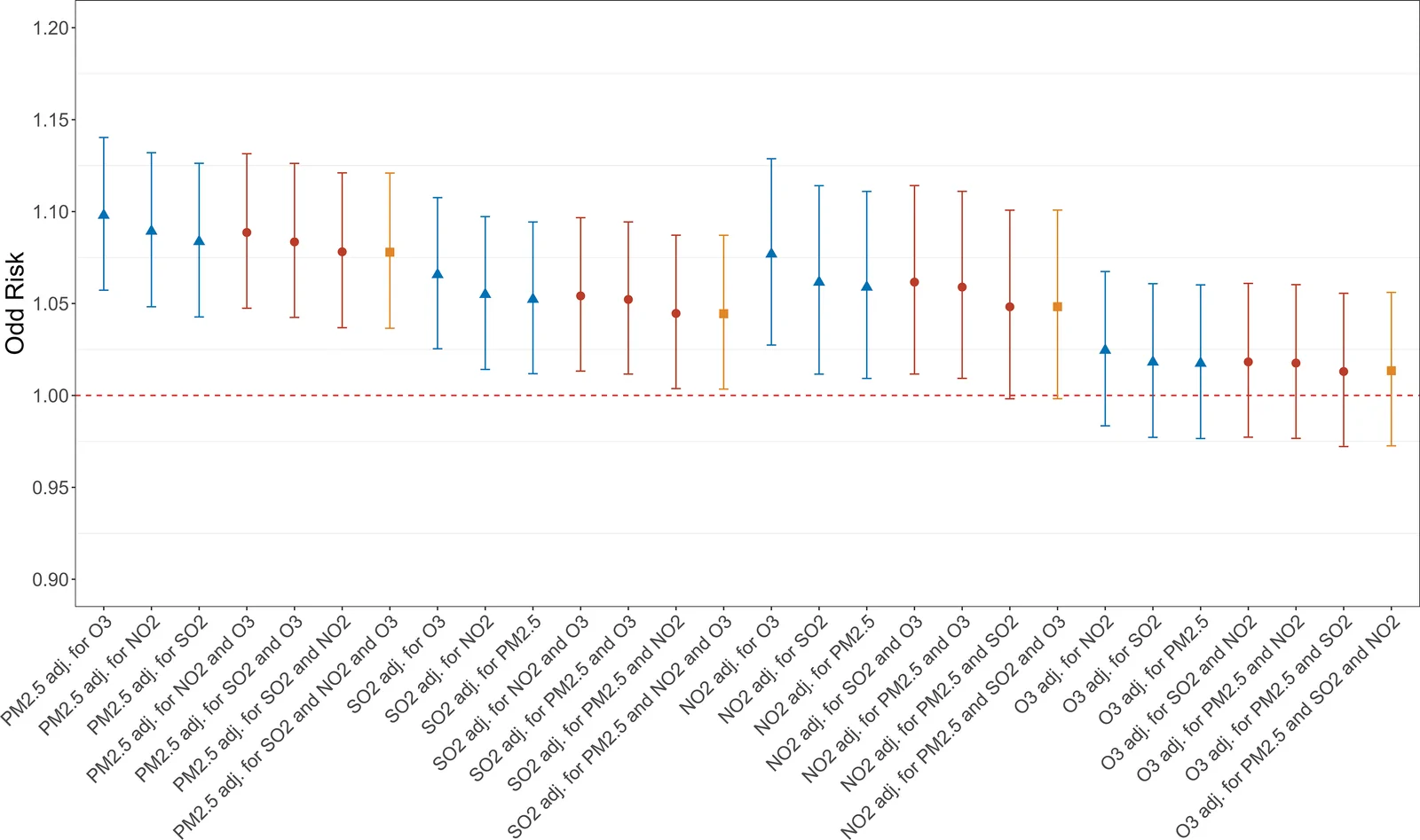
ORs (95% CIs) for CKD mortality in exposure to four air pollutants estimated by multi-pollutant models using conditional logistic regression models, adjusting for temperature and relative humidity

## Discussion

This study, drawing on mortality surveillance data from the Zhejiang Provincial Center for Disease Control and Prevention and air pollutant exposure data, conducts an in-depth investigation into the relationship between short-term exposure to air pollutants and the mortality risk of chronic kidney disease. Our findings not only identify the lag effects of different air pollutants (PM_2.5_, SO_2_, O_3_, and NO_2_) but also reveal their potential interactions and the role of subgroups. The findings of this study provide evidence-based guidance for public health departments and relevant policymaking bodies to develop targeted interventions. This holds significant importance for alleviating the burden of chronic kidney disease, reducing pressure on regional healthcare systems, and ultimately improving local health outcomes and quality of life.

The results of this study indicate that all investigated air pollutants increase the risk of CKD mortality. After adjusting for the influence of other pollutants, the impact of PM_2.5_ and SO_2_ on CKD mortality risk remained significant, suggesting that these two pollutants may have independent mechanisms for causing kidney damage. Our epidemiological findings align with existing literature while further expanding upon the current body of evidence. Prior research has indicated that long-term exposure to SO_2_ and PM_10_, NO_2_ is significantly associated with mortality among patients with end-stage renal disease [20]. However, our study uniquely demonstrates the robust and independent acute effects of SO_2_ and PM_2.5_ on CKD-related mortality. Research has shown that PM_2.5_ and SO_2_ can induce endothelial dysfunction, leading to vasoconstriction, increased blood pressure, and elevated pressure in renal vessels [21]. This subsequently reduces effective blood flow to the kidneys, further impairing renal function. Furthermore, these two pollutants can also induce inflammation and oxidative stress responses in the body, causing direct or indirect damage to the kidneys [22]. PM_2.5_, along with adsorbed heavy metals and organic compounds, can activate the immune system, leading to the release of inflammatory mediators (such as C-reactive protein and IL-6). These mediators can damage glomerular and tubular cells, accelerating the decline of kidney function and the onset of kidney disease [23]. Research has also indicated that exposure to SO_2_ may lead to a decrease in the estimated glomerular filtration rate (eGFR) and an increase in urinary albumin excretion [24]. The results of this study are consistent with these pathophysiological processes and provide an important basis for public health interventions and relevant policy-making, thereby reducing the overall disease burden of CKD and alleviating the pressure on regional healthcare systems.

Our sensitivity analysis excluding the year 2022 further confirmed the robustness of our findings, while also revealing a slight attenuation in the effect estimates. This attenuation can be primarily explained by two factors. First, the massive COVID-19 outbreak in late 2022 caused extreme strain on the regional healthcare system. During this period, healthcare accessibility was severely compromised. Second, from a methodological perspective, excluding a full year of data inherently reduces the overall sample size and statistical power, naturally leading to wider confidence intervals and more conservative point estimates. Nevertheless, the persistence of consistent associations even after excluding the pandemic peak underscores that the detrimental impact of air pollution on CKD mortality is a robust phenomenon and not an artifact of the COVID-19 pandemic.

This study also identified a lag effect of 1 and 3 days for the association between air pollutant exposure and mortality risk in patients with CKD. This lag may reflect the time required for pollutants to induce renal injury through a series of pathophysiological processes after entering the body. Upon an individual’s exposure to air pollutants, the body initiates inflammatory and oxidative stress responses; however, the resulting damage to renal cells is not instantaneous but rather a gradual, cumulative process [22]. Furthermore, the reduction in effective renal blood flow caused by PM_2.5_ exposure also requires a sustained period to trigger a significant deterioration of renal function [21]. Therefore, preventive strategies for CKD must fully consider the impact of this lag effect. By initiating targeted interventions proactively and continuously monitoring the health status of the population, the risk of air pollution-related CKD mortality can be reduced more effectively.

Our subgroup analysis revealed the heterogeneous effect of air pollutant exposure on the mortality risk in patients with CKD. The most prominent finding was the seasonal difference: the association was only statistically significant during the cold season but not in the warm season. This phenomenon may be attributed to a combination of environmental and physiological factors. On the one hand, temperature inversions often occur in the cold season, which creates a stable atmospheric layer that impedes the dispersion of pollutants. This leads to higher near-ground pollutant concentrations, consequently increasing the actual exposure levels for the population [25, 26]. On the other hand, exposure to a cold environment can activate the sympathetic nervous system, causing peripheral vasoconstriction, elevated blood pressure, and increased heart rate, which may impair the kidney’s autoregulation and reduce renal blood flow [27].

Furthermore, we found that the associations between NO₂ and SO₂ exposure and CKD mortality risk were statistically significant among the unmarried population. Compared to married individuals, unmarried people may face greater psychological stress, less social support, and relatively unhealthier lifestyle habits (e.g., smoking, unbalanced diet). Previous studies have demonstrated that limited social networks and long-term psychosocial stress can exacerbate systemic chronic low-grade inflammation, rendering these individuals physiologically more vulnerable to the acute oxidative bursts triggered by ambient pollutants [28]. These subgroup findings suggest that future public health intervention strategies should specifically focus on the cold season and be directed toward vulnerable populations with less social support to achieve more precise and effective CKD risk prevention and control.

However, several limitations of this study should be acknowledged. First, exposure assessment was based on model-derived ambient pollutant concentrations at participants’ residential addresses, which served as proxies for actual personal exposure. This approach has inherent limitations as it does not account for individual mobility patterns or the amount of time spent indoors. Consequently, the lack of detailed information regarding indoor exposure levels and daily activity trajectories may have introduced potential exposure misclassification bias. Second, our study cohort experienced the outbreak of the COVID-19 pandemic, which could potentially influence mortality rates from chronic kidney disease.

In conclusion, this study utilized a robust time-stratified case-crossover design to provide compelling evidence that short-term exposure to PM_2.5_, SO_2_, O_3_, and NO_2_ is significantly associated with increased CKD mortality in Zhejiang Province. This approach effectively controlled for individual-level confounding factors while mitigating the influence of long-term trends and seasonal variations. Furthermore, our analysis included nearly all CKD deaths in Zhejiang Province from 2019 to 2022, and we analyzed air pollutant concentrations with high precision, ensuring the study’s representativeness and accuracy. These findings hold direct public health implications, highlighting the urgent need to establish stricter air quality standards and develop seasonal early warning systems, particularly for vulnerable populations with limited social support. Future research should prioritize more precise exposure assessments using personal wearable sensors and investigate the biological pathways linking acute pollutant surges to renal failure, thereby enhancing population resilience.

## Supplementary Information

Below is the link to the electronic supplementary material.

Supplementary Material 1: Supplementary Figure 1. Definition of exposure lag periods

Supplementary Material 2: Supplementary Figure 2. Flowchart of participant selection and exclusion

Supplementary Material 3: Supplementary Figure 3. ERs (95 % CIs) for CKD mortality associated with each IQR μg/m3 increase of exposures to four air pollutants during pre-COVID-19 pandemic (2019–2021) period. *The X-axis is the single lag days from lag0 to lag7, and the moving average lag days from lag 01 to lag 07

Supplementary Material 4: Supplementary Table 1. Correlation coefficients among air pollutants

Supplementary Material 5: Supplementary Table 2. Interaction of air pollutants on CKD mortality

## Data Availability

Data is available on request from the corresponding author.
