# Supplementary material for: Short-term exposure to ambient air pollutants increases the mortality risk of chronic kidney disease: a time-stratified case-crossover study in Zhejiang Province
Source: BMC Nephrol. 2026 Jul 21;27:600. doi: 10.1186/s12882-026-05103-9 (PMC13654209; doi:10.1186/s12882-026-05103-9)
Supplement: Supplementary file 1 [file 12882_2026_5103_MOESM4_ESM.docx]

| Pollutants | Correlation coefficients | | | | VIF |
| --- | --- | --- | --- | --- | --- |
|  | NO_2_ | SO_2_ | O_3_ | PM_2.5_ |  |
| NO_2_ | 1.00 | 0.44 | -0.24 | 0.65 | 2.13 |
| SO_2_ | 0.44 | 1.00 | 0.18 | 0.53 | 1.81 |
| O_3_ | -0.24 | 0.18 | 1.00 | -0.03 | 2.37 |
| PM_2.5_ | 0.65 | 0.53 | -0.03 | 1.00 | 2.19 |
