# Supplementary material for: Short-term exposure to ambient air pollutants increases the mortality risk of chronic kidney disease: a time-stratified case-crossover study in Zhejiang Province
Source: BMC Nephrol. 2026 Jul 21;27:600. doi: 10.1186/s12882-026-05103-9 (PMC13654209; doi:10.1186/s12882-026-05103-9)
Supplement: Supplementary file 2 [file 12882_2026_5103_MOESM5_ESM.docx]

|  | Low Level | High Level | P _interaction_ | RERI (95% CI) |
| --- | --- | --- | --- | --- |
| level of NO_2__lag03 | Low-level O_3__lag01 (4.41-94.07) | High-level O_3__lag01 (94.07-267.43) |  |  |
| Low (4.23-25.77) | 1.00(Ref.) | 0.99(0.94,1.04) | 0.035 | -0.70(-0.83, -0.57) |
| High (25.77-108.57) | 1.04(0.99,1.10) | 1.12(1.05,1.19) |  |  |
| level of SO_2__lag02 |  |  |  |  |
| Low (2.05-7.84) | 1.00(Ref.) | 1.05(1.00,1.10) | 0.198 | -0.71(-0.83, -0.58) |
| High (7.84-35.89) | 1.11(1.06,1.17) | 1.09(1.03,1.15) |  |  |
| level of PM_2.5__lag02 |  |  |  |  |
| Low (3.34-23.82) | 1.00(Ref.) | 1.06(1.01,1.12) | 0.051 | -0.77(-0.90, -0.64) |
| High (23.82-215.60) | 1.16(1.10,1.22) | 1.13(1.07,1.19) |  |  |
| level of O_3__lag01 | Low-level PM_2.5__lag02 (3.34-23.82) | High-level PM_2.5__lag02 (23.82-215.60) |  |  |
| Low (4.41-94.07) | 1.00(Ref.) | 1.15(1.09,1.20) | 0.051 | -0.74(-0.85, -0.63) |
| High (94.07-267.43) | 1.05(1.00,1.10) | 1.11(1.05,1.17) |  |  |
| level of NO_2__lag03 |  |  |  |  |
| Low (4.23-25.77) | 1.00(Ref.) | 1.00(0.95,1.06) | <0.001 | -0.72(-0.83, -0.61) |
| High (25.77-108.57) | 0.97(0.91,1.03) | 1.14(1.08,1.21) |  |  |
| level of SO_2__lag02 |  |  |  |  |
| Low (2.05-7.84) | 1.00(Ref.) | 1.05(0.99,1.10) | 0.024 | -0.72(-0.83, -0.62) |
| High (7.84-35.89) | 0.99(0.94,1.05) | 1.13(1.08,1.19) |  |  |
| level of O_3__lag01 | Low-level SO_2__lag02 (2.05-7.84) | High-level SO_2__lag02 (7.84-35.89) |  |  |
| Low (4.41-94.07) | 1.00(Ref.) | 1.09(1.03,1.14) | 0.198 | -0.69(-0.80, -0.59) |
| High (94.07-267.43) | 1.03(0.99,1.08) | 1.07(1.01,1.12) |  |  |
| level of NO_2__lag03 |  |  |  |  |
| Low (4.23-25.77) | 1.00(Ref.) | 0.96(0.91,1.01) | <0.001 | -0.71(-0.82, -0.59) |
| High (25.77-108.57) | 0.99(0.94,1.05) | 1.13(1.07,1.19) |  |  |
| level of PM_2.5__lag02 |  |  |  |  |
| Low (3.34-23.82) | 1.00(Ref.) | 0.99(0.94,1.05) | 0.024 | -0.76(-0.87, -0.64) |
| High (23.82-215.60) | 1.05(0.99,1.10) | 1.13(1.08,1.19) |  |  |
| level of O_3__lag01 | Low-level NO_2__lag03 (4.23-25.77) | High-level NO_2__lag03 (25.77-108.57) |  |  |
| Low (4.41-94.07) | 1.00(Ref.) | 1.04(0.98,1.10) | 0.035 | -0.77(-0.87, -0.67) |
| High (94.07-267.43) | 0.98(0.94,1.03) | 1.09(1.03,1.16) |  |  |
| level of SO_2__lag02 |  |  |  |  |
| Low (2.05-7.84) | 1.00(Ref.) | 1.00(0.95,1.06) | <0.001 | -0.74(-0.84, -0.65) |
| High (7.84-35.89) | 0.95(0.90,1.00) | 1.10(1.04,1.17) |  |  |
| level of PM_2.5__lag02 |  |  |  |  |
| Low (3.34-23.82) | 1.00(Ref.) | 0.97(0.92,1.04) | <0.001 | -0.80(-0.90, -0.70) |
| High (23.82-215.60) | 0.99(0.94,1.04) | 1.12(1.06,1.19) |  |  |

*RERI: excess risk due to interaction
